# Subacute thyroiditis complicated with multiple organ failure: A case report

**DOI:** 10.1097/MD.0000000000028710

**Published:** 2022-02-04

**Authors:** Hui Jiang, Xiaoluo Chen, Li Wang, Xiaoqin Qian, Yan Zhang, Jing Wu, Shihe Shao

**Affiliations:** a Department of Endocrinology, Affiliated People's Hospital of Jiangsu University, 8 Dianli Road, Zhenjiang, Jiangsu Province, China; b Department of Ultrasound, Affiliated People's Hospital of Jiangsu University, 8 Dianli Road, Zhenjiang, Jiangsu Province, China; c School of Medicine, Jiangsu University, 301 Xuefu Road, Zhenjiang, Jiangsu Province, China.

**Keywords:** case report, multiple organ failure, subacute thyroiditis

## Abstract

**Rationale:**

Subacute thyroiditis is an inflammatory disease of the thyroid gland that is often caused by viral infections. Multiple organ failure (MOF) is mainly caused by acute inflammatory reactions resulting from severe infection or trauma. MOF due to subacute thyroiditis is extremely rare.

**Patient concerns:**

A 48-year-old woman with a history of type 2 diabetes mellitus was admitted to our hospital because of subacute thyroiditis. However, the patient developed MOF during hospitalization.

**Diagnosis:**

The patient was diagnosed with subacute thyroiditis complicated by MOF based on clinical symptoms and laboratory tests.

**Interventions:**

The patient was initially admitted to the endocrinology ward for glucocorticoid and insulin therapies. When the condition deteriorated to MOF, the patient was transferred to the intensive care unit. Ventilator-assisted breathing, blood transfusion, albumin infusion, improved cardiac function, oral glucocorticoids, and insulin were administered to the patient.

**Outcomes:**

The patient was followed-up at 2-weeks intervals for over 2 months. Her thyroid function returned to normal and her blood sugar level was stable. Transaminase, serum creatinine, albumin, and myocardial enzyme levels were normal.

**Lessons:**

MOF due to subacute thyroiditis is extremely rare. Especially in patients with elevated blood glucose or other immune dysfunctions, we should be alert to the occurrence of subacute thyroiditis with MOF.

## 1 Introduction

Subacute thyroiditis was first described in 1940, but its pathogenesis remains unclear. As most patients have prodromal symptoms of upper respiratory tract infection, it is believed that the occurrence of subacute thyroiditis is related to viral infection, which can directly destroy follicular cells and lead to inflammation.^[1]^ Multiple organ failure (MOF) refers to the syndrome of 2 or more organ dysfunctions or even functional failure after the organism is subjected to serious damage (such as serious disease, trauma, surgery, infection, and shock). The pathogenesis of MOF is complex, and most people believe that when the body is attacked, a systemic self-destructive inflammatory storm occurs, leading to MOF. Subacute thyroiditis is often considered a self-limiting disease and is complicated by MOF, which is extremely rare.

## 2 Case presentation

**Figure 1:**
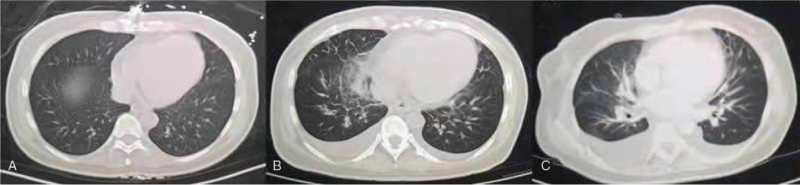
Dynamic changes of chest CT examination. (A) Chest CT examination at 8:00 am on November 17, 2021 revealed no significant lung abnormalities. (B) Chest CT examination at 5:39 am on November 22, 2021 revealed pulmonary edema in both lungs and a small pleural effusion. (C) CT re-examination at 11:30 am on November 22, 2021 showed progressive pulmonary edema, atelectasis of the right lower lung. CT = computed tomography.

A 48-year-old woman was admitted to our hospital on September 17, 2021, with intermittent neck pain for 3 weeks. There was no fever at the early stage of the disease, and she went to a community hospital and received cephalosporin antibiotic treatment; however, the pain was not relieved. On September 7, 2021, the woman underwent thyroid color Doppler examination, which revealed a patchy hypoechoic thyroid area and possible subacute thyroiditis. Routine blood examination showed a white blood cell count of 11.6 × 10^9^/L; neutrophils 76%; C-reactive protein 7.3 mg/L, hemoglobin 94 g/L; erythrocyte sedimentation rate 59 mm/h; and COVID polymerase chain reaction test results were negative. Thyroid function tests showed a low thyroid-stimulating hormone (TSH) of 0.398 uIU/mL (0.27–4.2 uIU/mL) and elevated free thyroxine of 21.88 pmol/L (7.64–16.03 pmol/L), and thyroid iodine uptake rate was significantly lower than normal. She was diagnosed with subacute thyroiditis, and prednisone (10 mg) was administered. Neck pain was relieved. Due to the rising trend of blood glucose, the patient stopped using prednisone on the night of September 16, 2021, and next morning, the patient developed aggravation of neck pain, the pain radiating to the retrosternal bone, accompanied by chills, fever, temperature as high as 39°C, nausea, vomiting, palpitation, and chest tightness. The patient was admitted to the emergency medical department of our hospital. After intravenous administration of 5 mg, her symptoms improved, and she was admitted to the endocrinology ward for further treatment. The patient had a 10-year history of type 2 diabetes. She had been taking glipizide combined with metformin for hypoglycemic treatment, and her blood glucose control was good in the past. Blood glucose monitoring during prednisone administration showed that the fasting blood glucose level was between 12 and 13 mmol/L, and the postprandial blood glucose was between 17 and 18 mmol/L. Physical examination: temperature 37.8°C, pulse 117 times/min, respiratory rate 18 times/min, blood pressure 140/80 mm Hg, right lobe of thyroid gland II degree enlargement, hard and tender, and heart rate 117 times/min. The lung and abdominal examinations were normal. Fine needle biopsy of the thyroid nodules revealed follicular epithelial cells, lymphocytes, and neutrophils. Echocardiography revealed a left ventricular ejection fraction of 54%. Electrocardiography revealed slight ST-segment depression. Color Doppler echocardiography of the carotid arteries showed intimal thickening with plaques. Chest computed tomography (CT) revealed no obvious abnormality (Fig. 1A). The patient was diagnosed with subacute thyroiditis and type 2 diabetes mellitus. Prednisone (10 mg) was administered as an anti-inflammatory, propranolol 10 mg TID to slow heart rate, aspirin for anti-platelet aggregation, atorvastatin for plaque stabilization, and insulin aspartic 30 for hypoglycemic injection before breakfast and dinner were administered. On the third day after admission, the patient developed aggravated neck pain and fever, with a temperature of 38.0°C, accompanied by nausea and vomiting. Omeprazole was administered to inhibit the gastric acid secretion. The patient's symptoms were not alleviated, and chest tightness gradually developed and the patient could not be supine. Chest CT revealed pulmonary edema in both lungs and a small pleural effusion (Fig. 1B). Blood gas analysis showed a buffer excess of –5.6, blood oxygen saturation of 83%, and no significant change in the electrocardiogram. Oxygen inhalation and furosemide diuresis were administered and the patient's symptoms abated for a short time. CT reexamination 6 hours later showed progressive pulmonary edema, atelectasis of the right lower lung, thickening and edema of the gallbladder wall, and pelvic effusion (Fig. 1C). Laboratory tests revealed several abnormalities, including elevated levels of transaminase, creatinine, brain natriuretic peptide, and myocardial enzymes, and low levels of oxygen saturation, albumin, hemoglobin (Table 1). Due to the rapid admission, the patient was transferred to the intensive care unit (ICU) and received intermittent non-invasive ventilation for assisted respiration. Digoxin and furosemide were administered to improve cardiac function, betaloc-reduced myocardial oxygen consumption, low molecular weight heparin anticoagulation, aspirin combined with clopidogrel/anti-platelet aggregation, trimetazine nourishes the myocardium, and prednisone modulates immunity. Hepatoprotective effects of magnesium isoglycyrrhizinate. Plasma albumin was injected and thoracic drainage was performed. The patient's neck pain gradually subsided, chest distress and asthma symptoms improved, diet returned to normal, blood sugar was stable, and lab parameters gradually recovered (Table 1).

**Table 1:** Laboratory value.

Test term	September 18, 2021	September 22, 2021	September 23, 2021	September 25, 2021	September 29, 2021	October 7, 2021	October 26, 2021	November 1, 2021
FT3 (3.28–6.47 pmol/L)	4.79	NA	6.43	NA	5.87	4.91	5.48	4.31
FT4 (7.64–16.03 pmol/L)	37.38	NA	65.41	NA	58.99	29.58	17.23	14.23
TSH (0.56–5.91 uIU/mL)	0.05	NA	0.04	NA	0.01	0.01	0.02	1.58
ESR (0–26 mm/h)	50	NA	NA	58	NA	NA	40	23
WBC (3.5–9.5 10^9^/L)	NA	NA	10.75	NA	NA	9.73	6.54	5.56
Hb (110–130)	NA	NA	76	NA	NA	NA	124	118
Alb (35–55 g/L)	30	NA	23	NA	26	31	34	35
AST (0–40 u/L)	36	67	287	829	56	45	40	42
ALT (0–40 u/L)	13	44	1312	1201	396	101	45	38
Cr (35–135 umol/)	78	NA	147	NA	81	68	51	46
BNP (0–110 pg/mL)	NA	NA	13866	NA	8952	5276	1567	123
Troponin (0–0.10 ng/mL)	0.21	3.28	18.35	2.62	0.59	0.48	0.07	
D-dimer (0–1.0 mg/L)	3.2	NA	15.5	NA	5.2	1.0	0.7	
LDH (110–220 u/)	193	NA	2289	563	59	225	214	

Alb = plasma albumin, ALT = glutamic-pyruvic transaminase, AST = glutamic oxalacetic transaminase, BNP = brain natriuretic peptide, Cr = creatinine, ESR = erythrocyte sedimentation rate, FT3 = free triiodothyronine, FT4 = free thyroxine, Hb = hemoglobin, LDH = lactate dehydrogenase, NA = not available, TSH = thyroid stimulating hormone, WBC = white blood cell.

After 2 weeks of treatment in the ICU, the patient was transferred to the general ward and continued to receive insulin aspartic 30 for hypoglycemia, prednisone 10 mg BID for subacute thyroiditis, and to reduce myocardial oxygen consumption. The patient was discharged on October 10, 2021, and was diagnosed with subacute thyroiditis, acute left heart failure, acute respiratory failure, acute renal insufficiency, moderate anemia, hypoproteinemia, and type 2 diabetes. The patient was followed-up regularly after discharge without neck pain, and thyroid function examination indicated that thyroid hormone levels had returned to normal (Table 1).

## 3 Discussion

Subacute thyroiditis is a self-limiting inflammatory disorder of the thyroid. The most common etiology of subacute thyroiditis is an inflammatory process caused by viral infection. The most common viruses that cause subacute thyroiditis are Coxsackie virus, influenza virus, echo virus, and mumps virus. COVID-19 has been reported to cause subacute thyroiditis.^[2]^ Subacute thyroiditis typically presents as neck pain, focal goiter, and thyrotoxicosis. Laboratory tests often show elevated thyroid hormone and decreased TSH levels, and ultrasonography may show focal or diffuse heterogeneous/hypoechogenic areas. The thyroid iodine uptake was low. In situations in which the diagnosis is doubtful, fine-needle aspiration would be helpful. Lymphocytes, mononuclear macrophages, and neutrophils may be detected in the puncture fluid smear.

In our case, the patient had suppressed TSH levels, elevated free thyroxine levels, and elevated inflammatory markers. Low radioactive iodine uptake and fine-needle aspiration were also performed to exclude suppurative thyroiditis, and the diagnosis of subacute thyroiditis was definite. Therapy for subacute thyroiditis involves symptomatic measures and prednisone administration. However, our patient's condition did not improve after treatment with prednisone and subsequently deteriorated, with MOF of the heart, liver, kidney, and lung, accompanied by severe anemia and hypoproteinemia. After ICU treatment, the patient's organ function gradually recovered.

Subacute thyroiditis generally has a good prognosis, and MOF is extremely rare. Regarding the causes of MOF in this patient, it was considered that the patient had a history of type 2 diabetes for many years, and the blood glucose level increased significantly after taking prednisone at the beginning, resulting in a systemic acute inflammatory storm caused by immune deficiency. Although the exact interaction between diabetes mellitus and viral infection is not well understood, host defense against viral infection is largely mediated by immunity and the synthesis of related cytokines, such as interleukins and interferons.^[3]^

Previous studies have shown that diabetes and its related conditions can downregulate the immune system by impairing the functions of innate immunity, such as chemotaxis, phagocytosis, and the activity of neutrophils and macrophages, leading to severe illnesses.^[4,5]^ In addition, the patient had a long course of diabetes, suggesting that she had a variety of chronic complications of diabetes, including macrovascular and microvascular lesions; therefore, it is rational to hypothesize that these chronic complications might increase poor outcomes of viral infection. Meanwhile, high level of thyroid hormone due to subacute thyroiditis can aggravate the cardiac load, increase myocardial ischemia, as well as it can cause hypoxia of liver cells and lead to abnormal liver function. Therefore, it can be considered that the combined action of these factors led to the occurrence of MOF in this patient.

Although subacute thyroiditis is self-limiting, when combined with diabetes or other immune function blemishes, there is the potential for MOF, and as a clinician, we need to be able to detect these changes over time. Therefore, intensive monitoring and antidiabetic therapy should be considered for patients with diabetes and viral infections.

## Acknowledgments

The authors thank the physicians and nurses of the affiliated People's Hospital of Jiangsu University Department of endocrinology and comprehensive ICU.

## Author contributions

Hui Jiang, Xiao-qin Qian, and Shi-he Shao designed the research and wrote the paper. Hui Jiang, Xiaoluo Chen, Yan Zhang, Li Wang, and Jing Wu were responsible for the case collection and data collection analysis. All authors read and approved the final manuscript.

**Data curation:** Yan Zhang.

**Funding acquisition:** Xiaoqin Qian.

**Investigation:** Jing Wu.

**Project administration:** Li Wang, Shihe Shao.

**Resources:** Xiaoluo Chen.

**Writing – original draft:** Hui Jiang.

**Writing – review & editing:** Hui Jiang, Xiaoqin Qian, Shihe Shao.
